# Clinical and laboratory characteristics of patients with symptomatic secondary immunodeficiency following the treatment of haematological malignancies

**DOI:** 10.1002/jha2.683

**Published:** 2023-04-01

**Authors:** Adrian M. Shields, Sian E. Faustini, Siobhan Young, Sarah Terjesen, Nicholas I. McCarthy, Rachel L. Anderson, Mark T. Drayson, Alex G. Richter

**Affiliations:** ^1^ Clinical Immunology Service Institute of Immunology and Immunotherapy University of Birmingham Medical School Birmingham UK; ^2^ Department of Clinical Immunology University Hospitals Birmingham NHS Foundation Trust Birmingham UK

**Keywords:** haematological malignancy, immunoglobulin replacement, secondary immunodeficiency, vaccination

## Abstract

Secondary immunodeficiency (SID), manifesting as increased susceptibility to infection, is an emergent clinical problem in haematoncology. Management of SID includes vaccination, prophylactic antibiotics (pAbx) and immunoglobulin replacement therapy (IgRT). We report clinical and laboratory parameters of 75 individuals, treated for haematological malignancy, who were referred for immunological assessment due to recurrent infections. Forty‐five were managed with pAbx while thirty required IgRT after failing to improve on pAbx. Individuals requiring IgRT had significantly more bacterial, viral and fungal infections resulting in hospitalization at least 5 years after their original haemato‐oncological diagnosis. Following immunological assessment and intervention, a 4.39‐fold reduction in the frequency of hospital admissions to treat infection was observed in the IgRT cohort and a 2.30‐fold reduction in the pAbx cohort. Significant reductions in outpatient antibiotic use were also observed in both cohorts following immunology input. Patients requiring IgRT were more hypogammaglobulinaemic and had lower titres of pathogen‐specific antibodies and smaller memory B cell populations than those requiring pAbx. Test vaccination with pneumococcal conjugate vaccine discriminated poorly between the two groups. Patients requiring IgRT could be distinguished by combining wider pathogen‐specific serology with a frequency of hospital admissions for infection. If validated in larger cohorts, this approach may circumvent the need for test vaccination and enhance patient selection for IgRT.

## 1 INTRODUCTION

Symptomatic secondary immunodeficiency (SID) may be defined as an increased susceptibility to bacterial, viral or fungal infections arising from environmental factors (e.g. nutritional state) or other disease processes (e.g infection, inflammation, malignancy) and their treatments (e.g. cytotoxic or biologic chemotherapy).  Symptomatic SID is estimated to be 30‐fold more common than primary immunodeficiencies, but the epidemiology, risk factors and immunopathogenesis of symptomatic SID remain poorly understood [1].

The UK has observed a sustained increase in demand for immunoglobulin replacement therapy (IgRT) to manage patients with recurrent infections due to SID [2, 3], mainly from individuals previously treated for haematological malignancies. A growing armamentarium of biological and small molecule therapeutics is now employed to treat haematological malignancies leading to well‐documented improvements in overall survival [4, 5]. However, improvements in overall survival may lead to the emergence of clinically significant, long‐term immunocompromise in cancer survivors [1].

Strict demand management governs the use of IgRT in the United Kingdom. Hypogammaglobulinaemia (IgG < 4 g/L), a 6‐month trial of prophylactic antibiotics (pAbx) and failure to respond to pneumococcal vaccination are mandated in patients with recurrent infections prior to the initiation of IgRT for SID under previous and existing guidelines [6]. However, there is no evidence base guiding what pAbx should be employed, what constitutes a normal vaccination response in individuals with haematological or other comorbidities or how vaccine responses should be assessed [7]. Furthermore, the evidence supporting the use of IgRT in haematological malignancies derives from studies undertaken before the introduction of modern therapeutic strategies, most notably anti‐CD20 B cell depleting agents and CAR‐T cell therapy [8, 9, 10, 11], and prior to the institution of widespread childhood vaccination programmes that have impacted the incidence of invasive pneumococcal disease in adults [12].

The purpose of this study was to describe the clinical and immunological characteristics of a heterogeneous cohort of patients referred to Clinical Immunology for the assessment of recurrent infections following treatment for haematological malignancy. In describing this cohort of patients, we aimed to identify biomarkers that might differentiate individuals who might benefit from IgRT at the point of referral without subjecting them to unnecessary pAbx trial periods.

## 2 METHODS

A comprehensive, retrospective note review was conducted on all patients referred to the Immunodeficiency service at the Queen Elizabeth Hospital Birmingham from January 2014 to June 2019. Patients with a primary diagnosis of haematological malignancy and no prior diagnosis of primary immunodeficiency were included in this study. In total, data were collected on 75 consecutive patients including baseline immunoglobulin G, A and M concentrations, electrophoresis results, IgG serotype‐specific antibodies to tetanus toxoid, *Haemophilus influenzae* B (HiB), meningococcus serogroup C and pneumococcal serotypes 1, 3, 4, 5, 6B, 7F, 9 V, 14, 18C, 19A and 19F. Haematological parameters at referral were also recorded including haemoglobin concentration and lymphocyte, platelet and neutrophil counts. Lymphocyte subset and B lymphocyte immunophenotyping were determined by flow cytometry. A subset of patients was test‐vaccinated with pneumococcal conjugate vaccine 13 (PCV13); this was only undertaken if there was a reasonable expectation the patient would be able to respond (i.e not known to be B cell aplasic, at least 6 months since prior rituximab, or have complete panhypogammaglobulnaemic). All patients were HIV‐negative. The data presented in this study pertains to the immunological tests undertaken at the patients’ initial immunological assessment unless stated otherwise.

All immunological studies were undertaken by the University of Birmingham Clinical Immunology Service using assays accredited to the UK Accreditation Service 15189.2012 standards. Lymphocyte subset numbers were determined by using the BD TruCount method, and B memory lymphocyte phenotyping was performed using the EUROClass method as previously described on BD FACS Canto cytometers [13]. Naive B cells are defined as CD19+ IgD+ CD27‐, unswitched marginal‐zone‐like B cells as CD19+ IgD+ CD27+ and switched memory B cells as CD19+ IgD‐ CD27+. The lower limit for reliable determination of B lymphocyte subsets was set at 0.03 × 10^9^/L. Patients with clonal populations (e.g. chronic lymphocyte leukaemia) and those with B cell counts below the lower limit of reliable determination were excluded from flow cytometric analysis. Pathogen‐specific antibodies were determined using an in‐house Luminex immunoassay [14]. Seroprotective levels of IgG were defined as 0.10 IU/ml for tetanus, 1.0 μg/ml for HiB, 2.0 μg/ml for Meningococcus serogroup C, and 0.35 μg/ml for all pneumococcal serotypes. Total immunoglobulins were determined by nephelometry (Hitachi COBAS 6000, Roche). Where paraproteins were present, residual polyclonal immunoglobulin concentrations were calculated by subtraction of paraprotein concentration (estimated by densitometry) from the total IgG level (determined by nephelometry).

Clinical information regarding diagnosis, haematological treatment, hospital admissions, infection frequency and type, positive microbiological samples and antimicrobial use and radiological investigations were collated for each patient from the hospital records. The decision to initiate immunoglobulin replacement was made in accordance with the 2011 UK Department of Health Clinical Guidelines for Immunoglobulin Use, 2nd edition [15] or the 2019 Updated Commissioning Criteria for the use of therapeutic immunoglobulin (Ig) in immunology, haematology, neurology and infectious disease in England [6] depending on the time of assessment. All patients were followed up for at least 6 months after the initiation of treatment.

Data were analysed using a combination of GraphPad Prism Version 9 (GraphPad Software, San Diego California) and R studio [16] with principal component analysis facilitated by the tidyverse, ggfortify and cluster packages.

## 3 RESULTS

**FIGURE 1:**
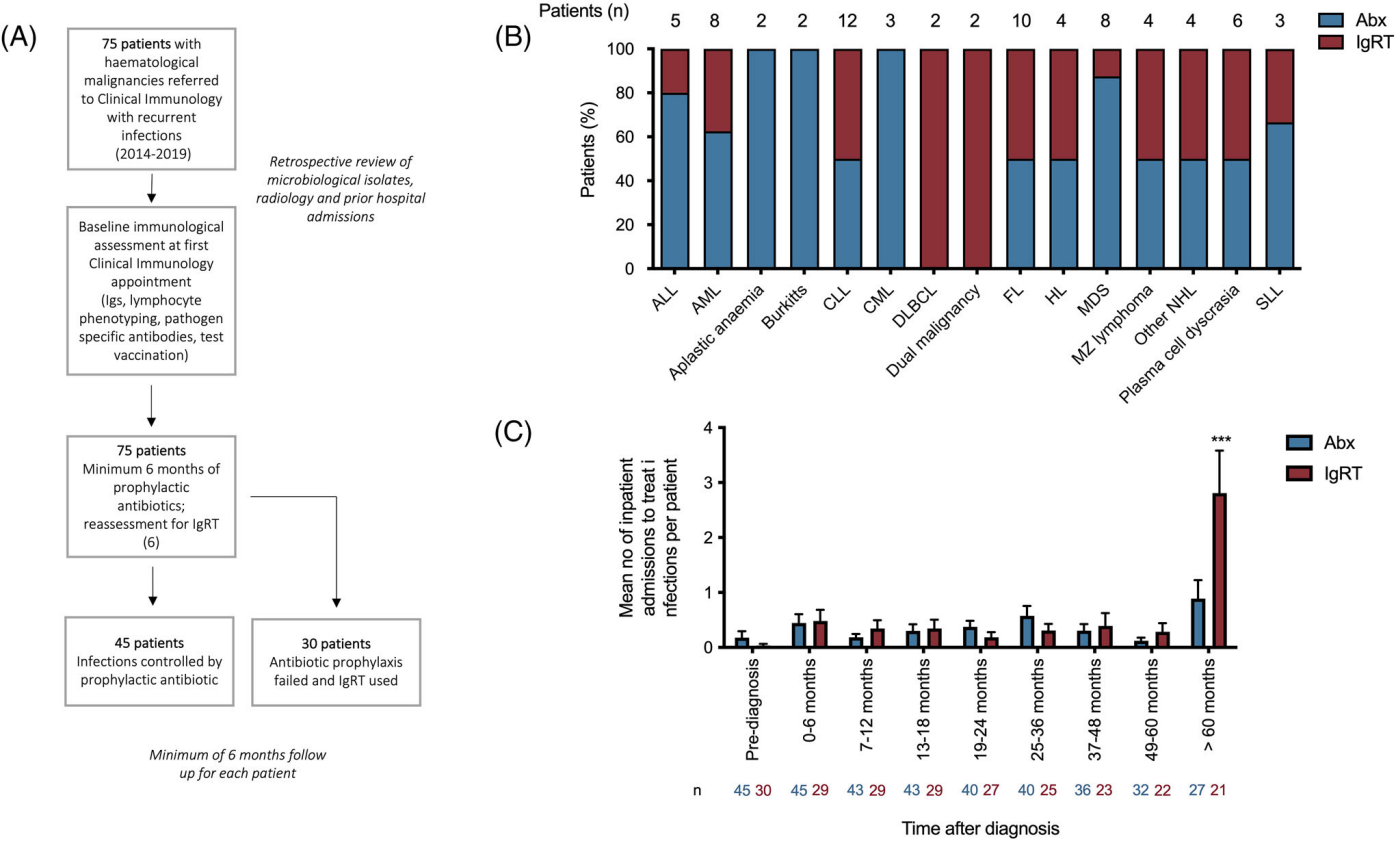
(A) Flow diagram of the assessment and management of patients in this study. (B) Underlying haematological diagnoses in 75 patients referred for investigation of recurrent infections between 2014–2019 and their immunological treatment prophylactic antibiotics (Abx) or immunoglobulin replacement therapy (IgRT). The number of patients with each diagnosis is provided above the individual columns. (C) The timing of hospital admissions to treat infections following the diagnosis of haematological malignancy. In each treatment group, the number of hospital admissions to treat infections occurring during each time period was documented. The number of individuals at risk is provided for each group provided below the bars. Data are expressed as the mean number of hospital admissions per patient per time period (e.g. between 49–60 months post‐diagnosis, individuals whose infections could be controlled using Abx alone suffered 0.12 infections requiring hospitalisation compared to 0.29 in the IgRT group). ***p < 0.0001 by two‐way analysis of variance (ANOVA).

Seventy‐five consecutive patients referred to Clinical Immunology for the investigation of recurrent infections between 2014 and 2019 are included in this study (Table 1). A flow diagram describing the assessment and management of these patients is provided in Figure 1A. The haematological and immunological laboratory data presented herein are those from the initial assessment with Clinical Immunology, prior to the initiation of IgRT. All patients ultimately requiring IgRT had used broad spectrum pAbx for at least 6 months which had failed to improve their recurrent infections. Forty‐five patients ultimately required IgRT and 30 remained stable on pAbx. No patients included within the pAbx group subsequently required initiation of IgRT. One patient included in the IgRT group successfully discontinued treatment following immune reconstitution and clinical improvement.

**TABLE 1:** Demographics and haematological parameters of patients with haematological malignancy associated secondary immunodeficiency.

	Abx	IgRT	*p*
*N*	45	30	
Age (Years) ‐ mean ‐ median ‐ range	55.4 64 20–77	59.1 63 30–88	NS
Sex (% female)	48.8	36.7	NS
HSCT (%) ‐ Allograft (%) ‐ Autograft (%)	55 88^$^ 12	40 83* 17**	NS
Time from first documented infection to immunology referral (Months) ‐ Mean ‐ Median	57.6 39.0	55.4 49.0	NS
Time under immunology follow‐up (months) ‐ Mean ‐ Median	29.3 25.0	38.0 40.50	0.023
Radiologically proven pneumonia prior to referral to immunology (%)	44.4	56.7	NS
Radiologically proven bronchiectasis prior to referral to immunology (%)	22.2	26.7	NS
Haematological parameters (mean, standard deviation) ‐ Hb (g/L) ‐ Platelet count (x10^9^/L) ‐ Neutrophil count (x10^9^/L) ‐ Lymphocyte count (x10^9^/L)	132.8 (23.1) 216.5 (83.6) 4.11 (2.18) 4.50 (13.10)	132.7 (19.9) 198.0 (106.1) 3.86 (2.16) 2.29 (2.94)	NS NS NS NS

* Not including one patient who had autograft followed by subsequent allograft.

** One patient also had two separate autografts.

^$^ One patient had a second allograft following initial graft failure.

Significance was determined by a two‐tailed Mann‐Whitney test for haematological parameters and Fisher's exact test for other demographics.

The underlying haematological diagnoses within the cohort are shown in Figure 1B. 49.3% of the patients had received a haematopoietic stem cell transplantation, the majority (85%) of which were allografts. The median delay between the first documented infection and referral to Immunology was 39.0 (interquartile range [IQR] 9.5–86.5) months for patients requiring pAbx and 49.0 (IQR 20.5–85.8) months for patients ultimately requiring IgRT.  By the time of referral, 49.3% of individuals had suffered at least one radiologically proven pneumonia and 24.0% of patients had radiological evidence of bronchiectasis. Patients were treated for a diverse range of infections prior to their referral to Clinical Immunology (Table 2). Infections characteristic of classical antibody deficiency (e.g. lower respiratory tract infections and sinusitis) occurred more frequently in patients requiring IgRT, but a significantly higher incidence of fungal pneumonia and non‐neutropenic sepsis/bacteraemia was also observed, suggesting broader, combined immune defects in these individuals, compared to those on pAbx. These patients also suffered significantly more late infections—defined as those occurring at least 5 years after their initial diagnosis of haematological malignancy—requiring hospital admission (Figure 1C).

**TABLE 2:** Treated infections in patients in inpatient and outpatient settings prior to referral to clinical immunology.

	Abx	IgRT	Rate ratio	Poisson Exact (95% CI)	*p*
*Respiratory tract*					
URTI	62	23	0.87	0.51–1.42	NS
Bacterial or viral LRTI or pneumonia	174	211	2.84	2.31–3.49	<2.2e‐16
Fungal LRTI or pneumonia	15	16	2.50	1.16–5.42	0.01
*Systemic*					
Febrile neutropenia/neutropenic sepsis	22	12	1.28	0.58–2.69	NS
Non‐neutropenic sepsis/bacteremia	6	19	7.41	2.85–22.68	0.000003
*Gastrointestinal*					
Gastroenteritis/colitis	9	9	2.34	0.82–6.56	NS
*Ear/nose/throat*					
Pharyngitis/Tonsilitis	3	1	0.78	0.01–9.72	NS
Otitis media	10	5	1.17	0.31–3.76	NS
Sinusitis	15	20	3.12	1.52–6.56	0.001
Oral candidiasis	7	6	2.01	0.56–6.97	NS
*Cutaneous*					
Skin/soft tissue infection	11	12	2.55	1.03–6.39	0.04
Fungal skin/nail infection	4	5	2.93	0.63–14.75	NS
Presumed HPV‐driven pathology (viral warts/CIN)	4	0	0.00	0.00–3.55	NS
*Genitourinary*					
UTI (including haemorrhagic cystitis)	12	18	3.51	1.60‐7.99	0.0009
*Other*					
Conjunctivitis/eye infection	3	4	3.12	0.53‐21.30	NS
*Overall*					
Any infection	**399**	**383**	**2.25**	**1.94‐2.59**	**< 2.2e‐16**

Only infections where four or more individual episodes across the whole cohort are shown. Presentations demonstrating a significantly greater incidence in the IgRT cohort are highlighted in red.

Abbreviations: CIN, cervical intraepithelial neoplasia; HPV, human papillomavirus; LRTI, lower respiratory tract infection; URT, upper respiratory tract infection.

A broad range of bacteria, viruses and fungi were identified from microbiological samples from this cohort (Table 3). Parainfluenza and respiratory syncytial virus were more likely to be detected in the group requiring IgRT; however, there was no difference in influenza detection. Non‐typable *Haemophilus influenzae* was isolated more frequently from patients requiring IgRT; however, there was no difference for *Streptococcus pneumoniae*, another encapsulated bacteria.

**TABLE 3:** Positive microbiological samples isolated from patients prior to referral to clinical immunology.

	Abx (*n* = 45)	IgRT (*n* = 30)	Incidence rate ratio	Poisson exact (95% CI)	*p*
*Viral isolates*					
CMV viraemia	9	3	0.78	0.14–3.12	NS
EBV viraemia	10	9	2.11	0.76–5.77	NS
Urinary BK virus	3	3	2.34	0.31–17.48	NS
Influenza A or B	5	6	2.81	0.71–11.63	NS
Parainfluenza (any type)	1	5	11.71	1.31–553.63	0.01
Respiratory syncytial virus	2	5	5.85	0.96–61.46	0.03
Herpes simplex virus 1 or 2	5	1	0.47	0.01–4.18	NS
*Bacterial isolates*					
Escherichia coli	4	3	1.76	0.26–10.37	NS
Staphylococcus aureus	2	4	4.68	0.67–51.76	0.06992
Streptococcus pneumonia	3	4	3.12	0.53–21.31	NS
Haemophilus influenza	5	11	5.15	1.65–18.91	0.002
Klebsiella sp.	0	4	–	1.55–Inf	0.008
Pseudomonas sp.	9	8	2.08	0.70–6.08	NS
Clostridium difficile	4	3	1.75	0.26–10.38	NS
*Fungal isolates*					
Candida species	7	6	2.01	0.56–6.97	NS
*Pneumocystits jiroveci*	4	2	1.17	0.11–8.17	NS
Overall					
All bacterial isolates	**29**	**40**	**3.23**	**1.95–5.40**	**0.000002**
All viral isolates	**40**	**39**	**2.28**	**1.43–3.64**	**0.0003**
All fungal isolates	14	9	1.50	0.57–3.73	NS

The incidence of microbiological isolates in each group is compared statistically using Poisson's incidence rate ratio (i.e. the total number of positive microbiological isolates occurring in each group corrected for the total time between the first infection and referral to clinical immunology in each group). Only pathogens where four or more individual isolates across the total cohort are shown).

57.3% of patients referred to Clinical Immunology were already taking at least one pAbx, which they had been receiving for a median of 39 months, the commonest being penicillin V, a narrow spectrum antibiotic commonly prescribed for presumed functional asplenia following transplant. The average number of pAbx employed prior to referral was not significantly different between those who required long‐term IgRT and those who did not (1.50 vs. 1.13, p = 0.264). Following referral to Clinical Immunology, the most commonly used pAbx was azithromycin and a much wider spectrum of pAbx prophylaxis was utilised to ensure infections could not be controlled with antibiotic prophylaxis alone (Figure S1).

Following assessment and intervention by Clinical Immunology, hospital admissions to treat infections significantly reduced; in the 45 individuals whose infections were ultimately controlled with pAbx, 23 hospital admissions to treat infections were observed in the year prior to immunological assessment and intervention compared to 10 in the following year (rate ratio 2.30; [confidence interval {CI} 1.05–5.41], *p* = 0.035). In the 30 individuals who ultimately needed IgRT to control recurrent infections, 52 hospital admissions to treat infection were observed in the year prior to immunological assessment and intervention compared to 12 in the following year (rate ratio 4.30 [CI 2.28–8.92], *p* < 0.0001). Reductions were also observed in the frequency of infections treated on an outpatient basis; pAbx group 90 vs 38 (rate ratio [CI 1.60–3.56, *p* < 0.00001]), IgRT group 89 vs 36, (rate ratio 2.47 [CI 1.66–3.75, *p* < 0.00001]).

**FIGURE 2:**
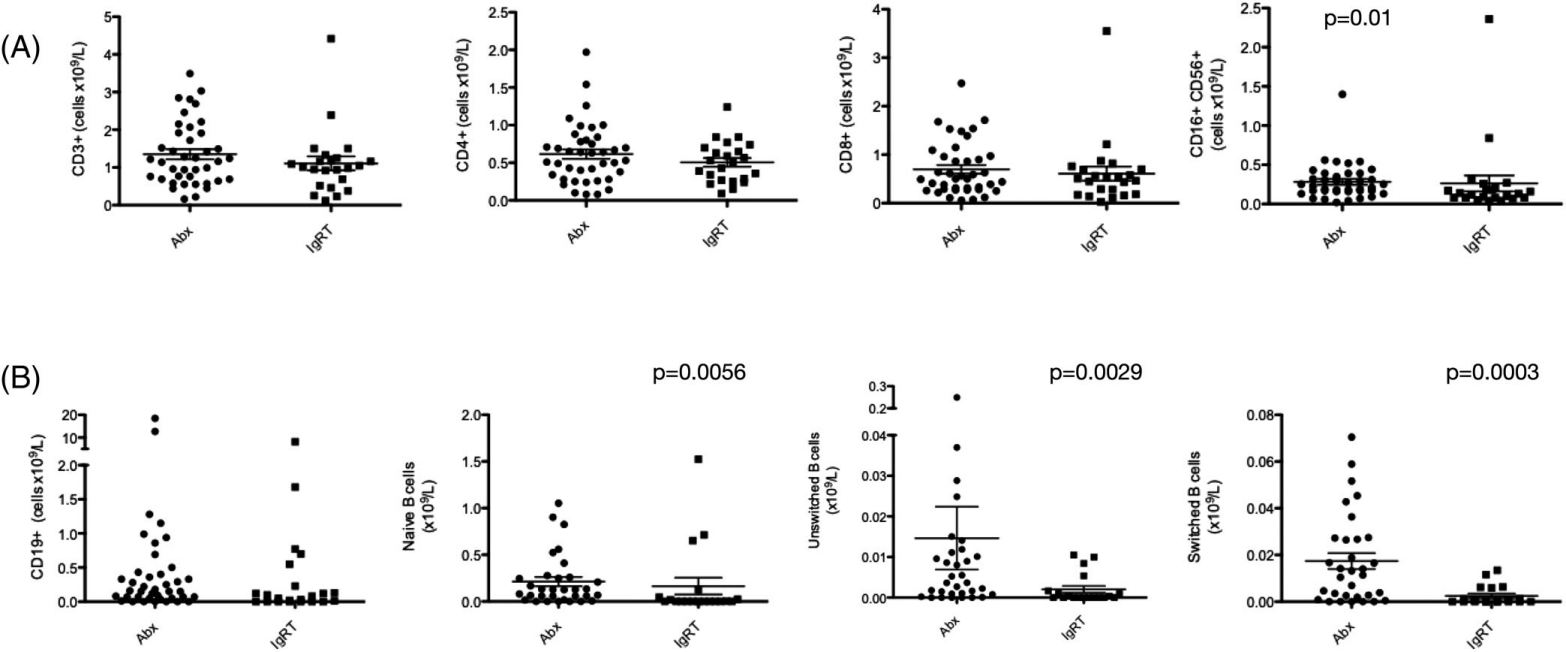
(A) Lymphocyte subset analysis in patients with symptomatic secondary immunodeficiency requiring treatment with prophylactic antibiotics (Abx) or immunoglobulin replacement therapy (IgRT). (B) CD19+ B cell phenotyping in patients with symptomatic secondary immunodeficiency. *p*‐Values are shown where statistical significance between the two groups was demonstrated using a two‐tailed Mann‐Whitney U‐test.

Haematological parameters including total neutrophil and lymphocyte counts did not discriminate between patients who could be maintained on pAbx and those requiring IgRT (Table 1). Enumeration of the total peripheral blood CD3+ T cell population and the CD4+ or CD8+ T cell subpopulations also did not discriminate between treatment groups (Figure 2A). 8% of the total cohort were found to have a CD4+ T cell count < 0.2 × 10^9^/L, a known risk factor for *Pneumocystis jiroveci* injection. A small, but statistically significant difference in the absolute size of the NK cell population (CD3‐ CD16+ CD56+) was observed; the clinical significance of this remains unclear. There was no significant difference in the overall size of the peripheral blood B cell population and no significant difference in the incidence of complete B cell aplasia between the two groups. However, the absolute size of the peripheral blood naive (CD19+ IgD+ CD27‐), unswitched memory (CD19+ IgD+ CD27+) and switched memory B cell population (CD19+ IgD‐ CD27+) were all significantly lower in patients requiring long‐term treatment with IgRT (Figure 2B).

**FIGURE 3:**
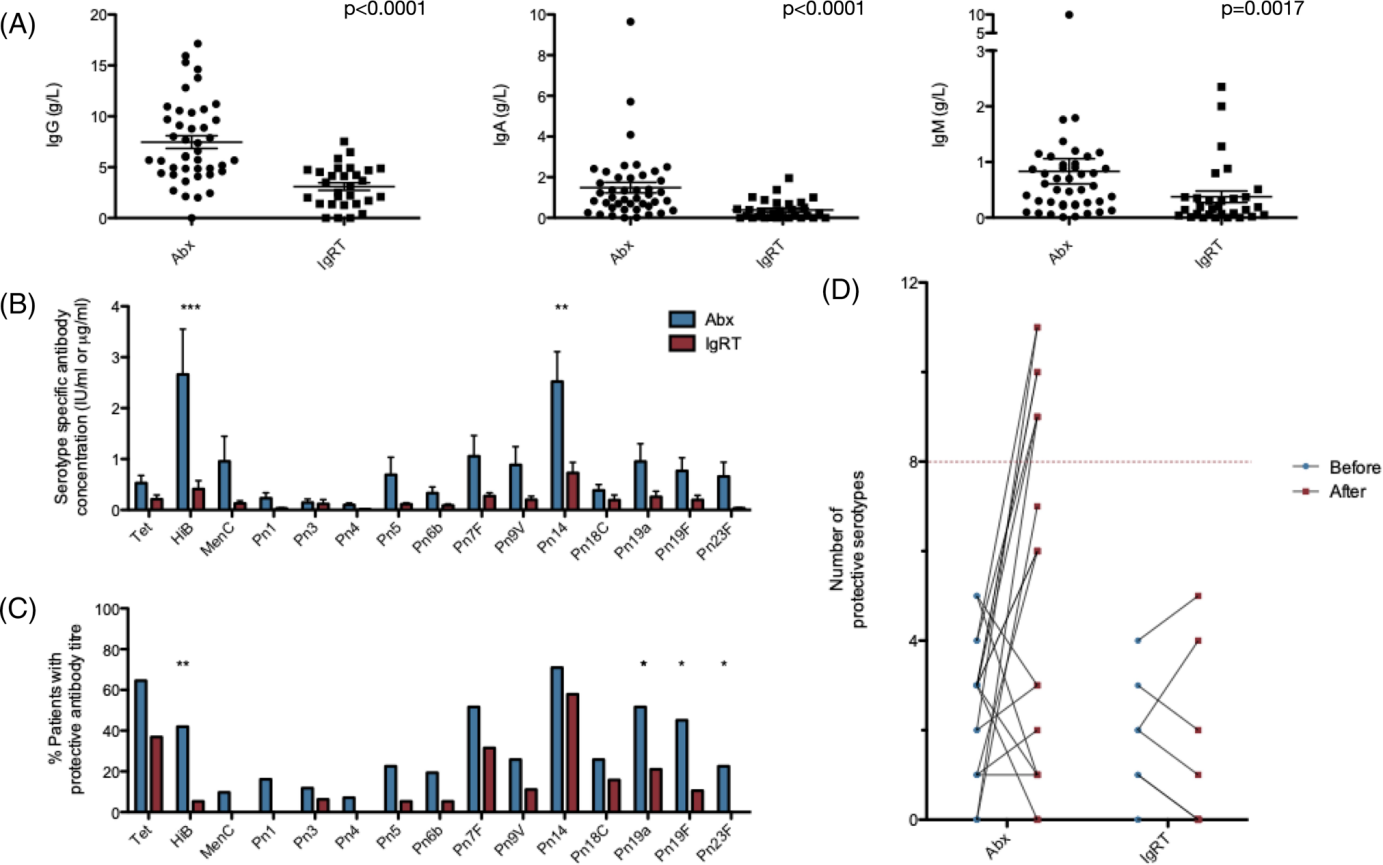
(A) Serum immunoglobulin G, A and M levels in patients with symptomatic secondary immunodeficiency requiring treatment with prophylactic antibiotics (Abx) or immunoglobulin replacement therapy (IgRT). *p*‐Values shown where statistical significance between the two groups was demonstrated using a two‐tailed Mann‐Whitney U‐test. (B) Baseline titres of pathogen‐specific antibodies amongst the cohort. (C) Percentage of patients demonstrating seroprotective titres of pathogen‐specific antibodies at baseline. (D) Response to vaccination with a 13‐valent pneumococcal conjugate vaccine in patients requiring Abx or IgRT. A normal response to vaccination is defined as achieving seroprotection in greater than 70% of measured serotypes (dotted red line). *p < 0.05, **p < 0.01, ***p < 0.001 by two‐way ANOVA.

As a cohort, patients requiring IgRT had lower total immunoglobulin G, A and M levels compared with those on pAbx (Figure 3A), however, overlap exists between the groups with no discriminatory level. 44% of patients in the pAbx group had an IgG level below the lower limit of the normal reference range (6 g/L) and 13% had an IgG level < 4 g/L, a level that would potentially make them eligible for immunoglobulin replacement for secondary antibody deficiency under the 2019 NHS England guidelines [6]. Equally, a small number of patients in the IgRT group had baseline IgG > 5 g/L but still had recurrent infections that had improved following the initiation of IgRT. There was no linear relationship between total IgG levels and the overall number of hospital admissions to treat infections prior to referral to Clinical Immunology (Spearman's *ρ* = 0.02, *p* = 0.86), or total IgG levels and the number of hospital admissions for late infections occurring 5 years after diagnosis of haematological malignancy (Spearman's *ρ* = −0.14, *p* = 0.24). There was also no linear relationship between the size of the peripheral switched memory B cell population and total serum IgG levels at the time of referral to Clinical Immunology (Spearman's *ρ* = 0.05, *p* = 0.69).

Functional humoral immunity against multiple pathogens including many common pneumococcal serotypes was poor in both treatment groups. Patients requiring long‐term IgRT had significantly lower absolute baseline titres of IgG directed against HiB and *Streptococcus pneumonia* serotype 14 (Figure 3B). Furthermore, the percentage of patients possessing seroprotective titres of antibodies against invasive disease caused by HiB, pneumococcal serotypes 19A, 19F and 23F was significantly lower in those requiring long‐term IgRT (Figure 3C). Response to test vaccination with pneumococcal conjugate vaccine 13 (PCV13) was also poor in both treatment groups; no patients in the IgRT and only 35% of patients in the antibiotic cohort mounted a satisfactory response to vaccination with PCV13, defined by an increment in protective serotypes to over 70% of those measured (Figure 3D).

**FIGURE 4:**
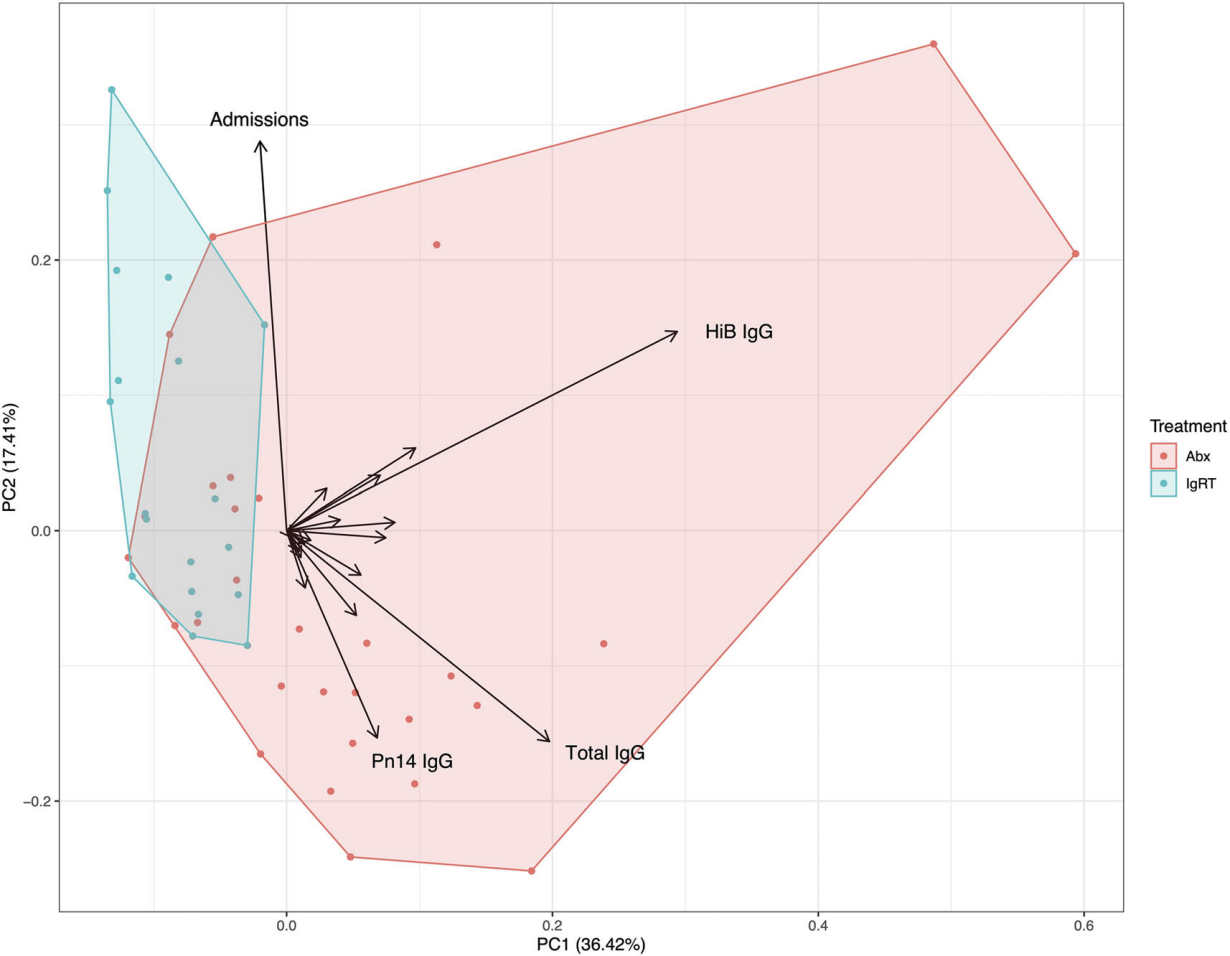
Unsupervised clustering (principal component analysis) of the two treatment groups based on baseline immunoglobulin G, A and M levels, hospital admissions to treat infections and functional IgG antibody titres directed against Tetanus toxoid, *Haemophilus influenzae* B, meningococcus serogroup C and pneumococcal serotypes. Eigenvectors for selected variables are labelled.

Current guidelines governing the use of immunoglobulin replacement therapy mandate patients undergo a minimum 6‐month trial of prophylactic antibiotics prior to initiation of IgRT. Patients requiring IgRT must therefore suffer further infectious morbidity before commencing potentially definitive therapy. We hypothesised that it may be possible to discriminate patients requiring IgRT from immunological and clinical parameters available at initial referral. Unsupervised clustering of the cohort was performed based on: baseline immunoglobulin G, A and M levels, the number of pre‐existing hospital admissions to treat infections and baseline functional antibody titres (Figure 4). Based on these simple parameters, two clusters emerged; however, due to the relatively small number of patients studied, overlap exists between them.

The cohort was divided into two subgroups; individuals who had undergone bone marrow transplantation and individuals who had not (Table S1). Mirroring the overall cohort, patients requiring long‐term IgRT in both subgroups had significantly lower baseline IgG and IgA levels. IgM levels were similar in patients who had not undergone bone marrow transplantation (Figure S2). Pathogen‐specific antibodies also showed similar patterns with both anti‐HiB and anti‐pneumococcal serotype 14 antibodies showing significant reductions in either absolute titre or the percentage of patients achieving a protective titre in the subgroups (Figure S2). Although the total lymphocyte count was significantly lower in patients who had undergone bone marrow transplantation and required long‐term IgRT, measurement of total T cells, the CD4 and CD8 T cell subsets or NK cells did not identify patients requiring long‐term IgRT in either subgroup. However, the incidence of complete B cell aplasia was greater in patients who had undergone bone marrow transplantation and who needed long‐term IgRT (44.4% vs. 8.7%, *p* = 0.04) (Figure S2).

**FIGURE 5:**
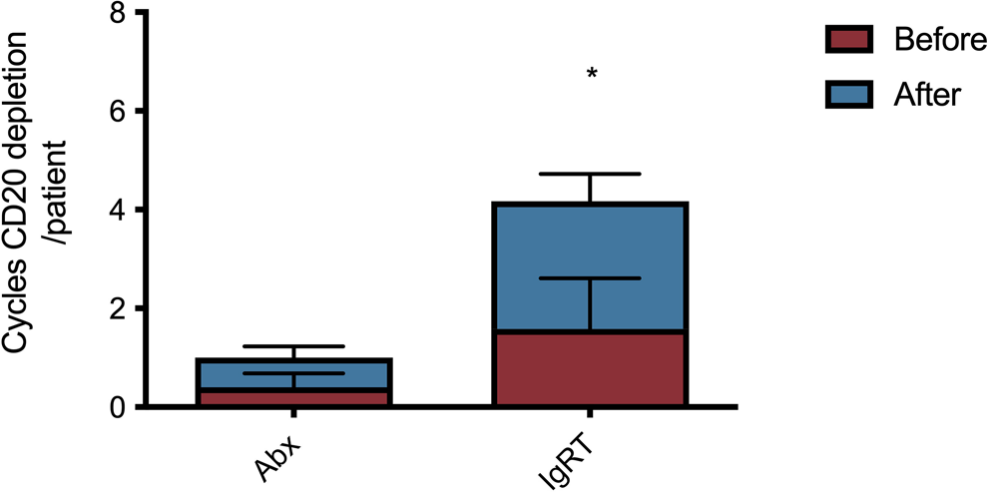
Mean number of cycles of B cell depleting agents before and after transplantation in patients who had received bone marrow transplantation. *p = 0.001, Mann‐Whitney's two‐tailed test.

We hypothesised that exposure to B cell depletion therapies may be a risk factor for persistent B cell aplasia and the requirement for long‐term IgRT in patients who had undergone bone marrow transplantation. Any exposure to anti‐CD20 B cell‐depleting agents in the bone marrow transplantation cohort was associated with an odds ratio of 10.63 (CI 1.87–60.2, *p* = 0.005) of requiring IgRT. Furthermore, the total exposure to anti‐CD20 B cell‐depleting agents was significantly higher in the patients requiring long‐term IgRT (Table S1). The timing of anti‐CD20 depletion may be important as we found patients requiring long‐term IgRT had significantly greater exposure to B cell depletion after transplantation (Figure 5) for indications including Epstein Barr virus reactivation and autoimmune cytopenias.

## 4 DISCUSSION

Increased susceptibility to infection following the diagnosis and treatment of haematological malignancy is predictable and well‐described. However, some individuals suffer recurrent late infections and it remains unclear whether pAbx or IgRT can impact morbidity or mortality. We provide a comprehensive description of a cohort of patients with symptomatic secondary immune deficiency following the treatment of haematological malignancy who have been assessed for consideration of IgRT.

Within this heterogeneous cohort, there is a subgroup of individuals who continue to suffer an increased frequency of severe infections requiring hospital admissions at least 5 years after their diagnosis of their haematological malignancy. Ultimately, these individuals appear to benefit from IgRT, as evidenced by the 4.30‐fold decrease in hospital admissions and a 2.47‐fold decrease in infections requiring outpatient treatment following initiation of treatment. The comparable rates of serious infections requiring hospital admission in the years immediately after diagnosis of haematological malignancy, likely directly related to pan‐immunosuppressive treatment, suggest these patients may be difficult to identify promptly using clinical parameters alone. Nevertheless, early identification and intervention for those who will develop a persistent secondary immunodeficiency are important to improve quality of life, prevent the development of bronchiectasis and potentially reduce healthcare costs by preventing recurrent hospital admissions.

Serum IgG concentrations and response to pneumococcal vaccination are currently used to stratify the severity of immunodeficiency and manage the demand for immunoglobulin products [6]. However, we show the significant limitations of these approaches. Although individuals requiring IgRT were significantly more hypogammaglobulineamic than those maintained using antibiotic prophylaxis, total IgG concentrations showed no correlation with the overall number of serious infections or late‐occurring serious infections requiring hospital admission. This is unsurprising as effective humoral immunity depends on the generation of high‐affinity, somatically hypermutated, class‐switched antibodies. Our data suggest that baseline pathogen‐specific antibodies may provide greater insight into this axis and the need for IgRT than total IgG or pneumococcal vaccine responsiveness.

We identify that absolute titres of IgG directed against pneumococcal serotype 14 and HiB are potential biomarkers of future IgRT requirements. Pneumococcal serotype 14 was, in the pre‐pneumococcal vaccine era, the most common cause of invasive pneumococcal infection worldwide [17]. Pneumococcal serotype 19A subsequently emerged as the most common cause of invasive pneumococcal disease following the introduction of the seven valent pneumococcal vaccines [18]; of note, the absence of a protective IgG titre to Pn19A was also associated with the need for IgRT in our cohort. Due to the age of our cohort, none would have benefited from childhood vaccination against HiB or pneumococcal disease; humoral immunity would have developed following infection or colonisation and be maintained by long‐lived plasma cells within the bone marrow [19, 20, 21]. The loss of humoral immunity to Pn14 and HiB may be a surrogate marker for the disruption of the reservoir of high‐quality humoral immunity and therefore provide valuable insight into the severity of the immune deficiency. These observations require wider validation in larger populations given the evolving landscape of pneumococcal disease and vaccination worldwide, and treatments that directly target the plasma cell pool including anti‐CD38 monoclonal antibodies. Importantly, when pathogen‐specific antibody levels were incorporated into a principal component analysis alongside total antibody levels and the number of hospital admissions, a distinct cluster of individuals who required IgRT emerged. This suggests a baseline serological assessment may identify patients who will maximally benefit from IgRT without the need for trials of prophylactic antibiotics or test vaccination. Notably, poor titres of pathogen‐specific antibodies have previously been associated with infection risk in individuals with CLL [22].

The response to test pneumococcal vaccination was blunted in almost all patients within this cohort. No individuals requiring IgRT and only 35% of individuals maintained on antibiotic prophylaxis mounted a satisfactory response to vaccination with a pneumococcal conjugate vaccine. These data suggest the diagnostic utility of pneumococcal vaccination in IgRT demand management may is limited in this cohort. Strikingly, the severe acute respiratory coronavirus syndrome 2 (SARS‐CoV‐2) pandemic has demonstrated that over 50% of individuals with profound antibody deficiencies are capable of mounting clinically effective vaccine responses to the initial two‐dose SARS‐CoV‐2 vaccination schedule, rising to 76.0% after three doses [23, 24, 25]. This raises the possibility of using alternative test vaccination strategies to interrogate immune competence and, that with better vaccine design and protocols, immune responses to existing non‐SARS‐CoV‐2 vaccines may be improved in immunocompromised cohorts. Furthermore, as pneumococcal polysaccharide vaccination becomes widely adopted in haemato‐oncology management guidelines [26], immunologists must be cognizant of the intersection between therapeutic vaccination and test vaccination, in particular, the potential for vaccine hyporesponsiveness through repeat exposure to pneumococcal vaccinations [27, 28].

The severity of secondary immunodeficiency is dynamic; we have previously demonstrated that kinetics of B cell reconstitution following anti‐CD20 therapy and its association with vaccine responses to SARS‐CoV‐2 vaccines differs between haematology and rheumatology cohorts as a factor of their underlying disease and other therapeutic immunosuppression [29]. In this cohort, we provide further evidence of the heterogeneity of the effects of B cell depletion on humoral immune competence. In the non‐bone marrow transplantation cohort of patients, total exposure to rituximab was not a significant risk factor determining the future requirements for IgRT, in keeping with rapid B cell reconstitution and restitution of vaccine responsiveness observed in most lymphoma patients following treatment [29]. However, in the bone marrow transplantation cohort, exposure to rituximab after transplantation was associated with a future need for immunoglobulin. Although rituximab use in this context is to treat Epstein Barr virus reactivation, viral reactivation may be a surrogate for a combination of poor immune reconstitution and/or increased immune suppression to treat graft versus host disease (GvHD). Aberrant B cell function is observed in chronic GvHD [30] and chronic GvHD has previously been identified as a risk factor contributing to late fatal infections in patients undergoing bone marrow transplantation alongside increasing age, male sex, and mismatched unrelated grafts [31]. The detrimental effect of these factors on the bone marrow plasma cell niche and consequent long‐term susceptibility to infection requires further research.

This study does have limitations: as a single‐centre experience conducted at a major bone marrow transplantation centre, transplant patients are likely to be over‐represented. Furthermore, the cohort is selected; we can only describe those patients who are referred for assessment because of recurrent infections. There remains no consensus on what constitutes recurrent infections significant enough to merit further immunological investigation during and after chemotherapy and so this is likely to vary nationally. Finally, we have analysed this cohort en masse, rather than in a disease‐specific manner; studies examining immune reconstitution in disease and treatment‐specific manner may help to better understand the relationships between responses to test vaccinations and susceptibility to severe infections. However, our study highlights the need for further studies to support the judicious use of immunoglobulin replacement in a growing cohort of survivors of haematological malignancy.

## AUTHOR CONTRIBUTIONS

Adrian M. Shields and Alex G. Richter conceived and designed the study. Adrian M. Shields, Alex G. Richter and Mark T. Drayson provided clinical care for the patients included in this study. Sian E. Faustini and Siobhan Young performed pathogen‐specific antibody studies, and Nicholas I. McCarthy and Sarah Terjesen performed flow cytometry studies. Rachel L. Anderson and Adrian M. Shields gathered clinical data for the study. Adrian M. Shields analysed the data, produced the first draft and revised the manuscript. All authors reviewed and commented on and approved the manuscript prior to submission.

## CONFLICT OF INTEREST STATEMENT

Mark T. Drayson reports personal fees from Abingdon Health, outside the submitted work. All other authors declare no competing interests.

## FUNDING INFORMATION

No specific funding was received for this study.

## ETHICS STATEMENT

This study reports fully anonymized, routinely collected data regarding patient outcomes. No specific ethical approval is required for collation or publication in accordance with UK NHS HRA guidance.

## Supporting information



Supporting Information

Supporting Information

Supporting Information

## Data Availability

An anonymized data set is available upon reasonable request from the corresponding authors.
