# Supplementary material for: Clinical and laboratory characteristics of patients with symptomatic secondary immunodeficiency following the treatment of haematological malignancies
Source: EJHaem. 2023 Apr 1;4(2):339–49. doi: 10.1002/jha2.683 (PMC10188475; doi:10.1002/jha2.683)
Supplement: Supplementary file 1 — Supporting Information [file JHA2-4-339-s001.docx]

**Supplementary Table 1**

* Not including one patient who had autograft following by subsequent allograft.

** One patient had a second allograft following initial graft failure

|  | Bone marrow transplantation patients | | | Non bone marrow transplantation patients | | |
| --- | --- | --- | --- | --- | --- | --- |
|  | Abx | IgRT | p | Abx | IgRT | p |
| N | 25 | 12 |  | 20 | 18 |  |
| Age (yrs) | 49.64 | 46.92 | NS | 59.1 | 63.6 | NS |
| Sex (%F) | 58.0 | 46.0 | NS | 45.0 | 44.4 |  |
| Allograft (%) | 88%** | 83%* | NS | n/a | n/a |  |
| % Mismatched Allograft  (HLA-A, B or C) | 4.3% | 30% | NS | n/a | n/a |  |
| Time from first infection to immunology referral (m) | 71.4 | 45.8 | NS | 40.5 | 61.8 | 0.02 |
| Time under immunology follow up (m) | 26.2 | 37.7 | 0.05 | 36.1 | 41.6 | NS |
| Hb (g/L) | 136.1 | 141.8 | NS | 132.1 | 126.6 | NS |
| Platelet count (x10^9^/L) | 236.8 | 150.8 | 0.003 | 171.6 | 229.5 | NS |
| Neutrophil count (x10^9^/L) | 4.51 | 3.92 | NS | 3.54 | 3.82 | NS |
| Lymphocyte count (x10^9^/L) | 2.29 | 1.62 | 0.02 | 7.51 | 2.77 | NS |
| Anti-CD20 B cell depletion  (cycles) | 25 | 50 | <0.001 | 179 | 122 | NS |

Significance determined by two-tailed Mann-Whitney test for haematological parameters and Fisher’s exact test for other demographics.
