# Supplementary figures and images for: Clinical and laboratory characteristics of patients with symptomatic secondary immunodeficiency following the treatment of haematological malignancies
Source: EJHaem. 2023 Apr 1;4(2):339–49. doi: 10.1002/jha2.683 (PMC10188475; doi:10.1002/jha2.683)

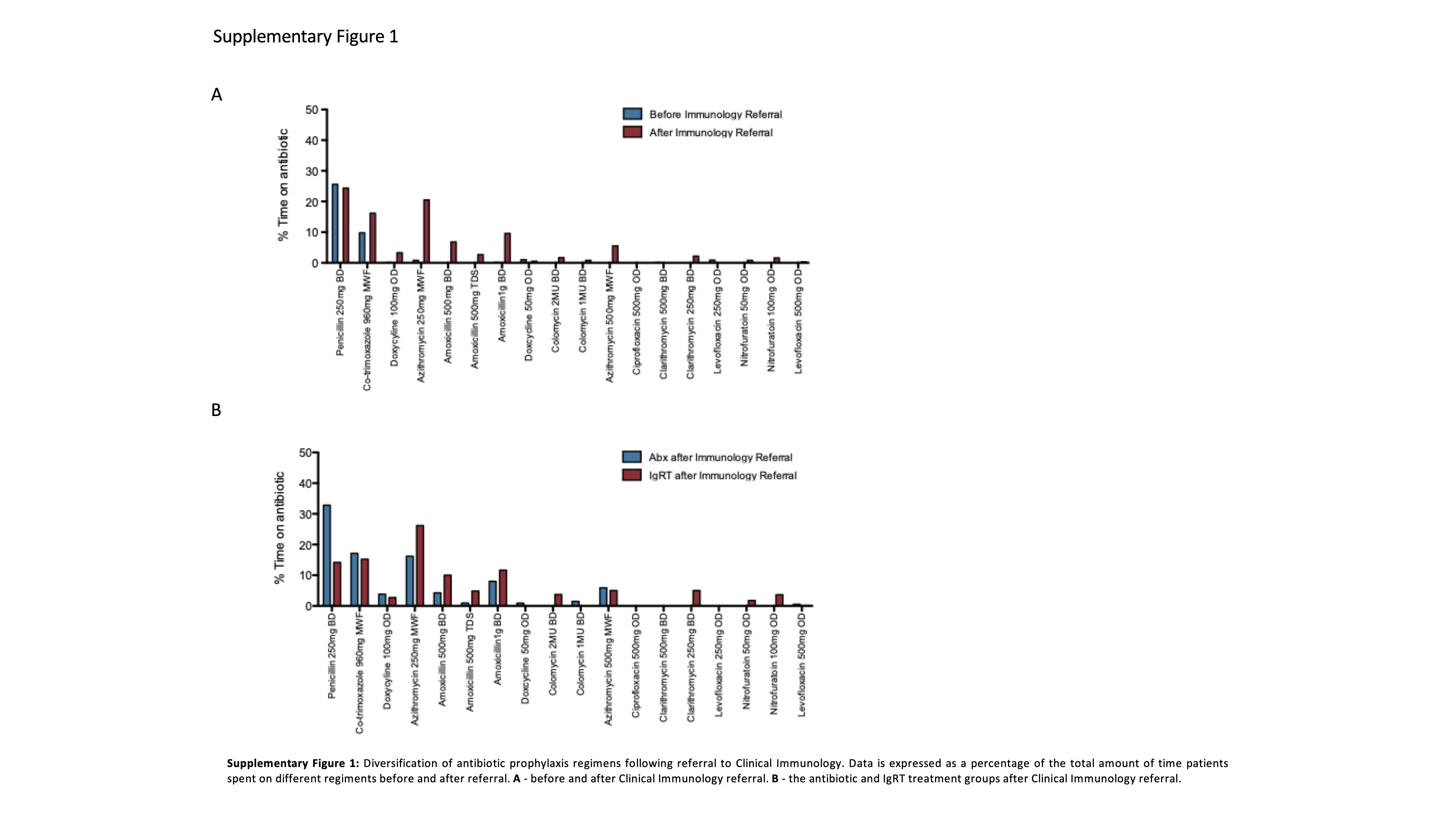

Supplement: Supplementary file 2 — Supporting Information [file JHA2-4-339-s002.jpg]

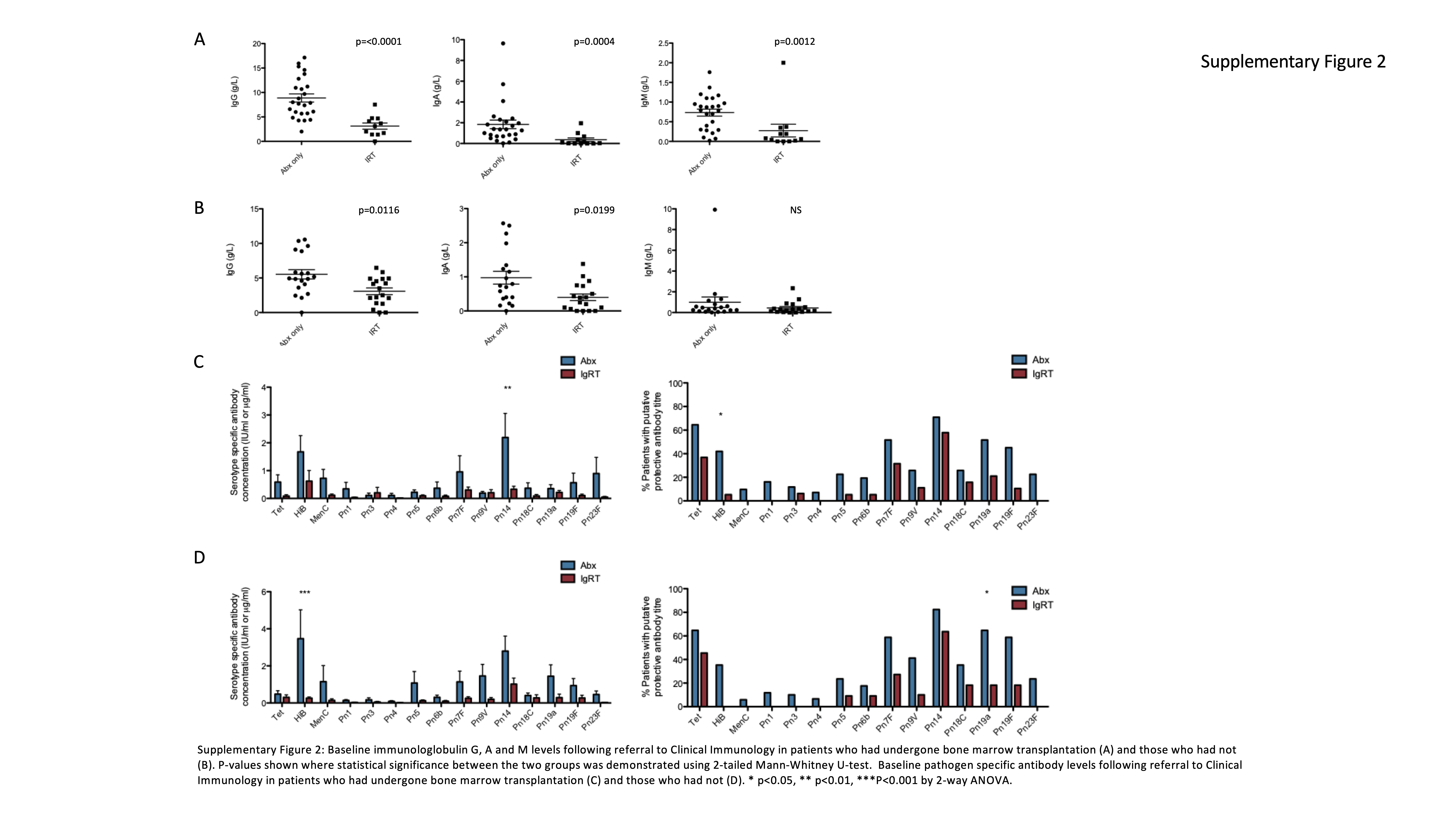

Supplement: Supplementary file 3 — Supporting Information [file JHA2-4-339-s003.jpg]
